# Reliability and validity of the QASCI questionnaire to assess caregiving burden in COPD

**DOI:** 10.1080/07853890.2021.1897441

**Published:** 2021-09-28

**Authors:** Nádia Hipólito, Adriana Ruivo, Sara Martins, Sofia Flora, Alda Marques, Dina Brooks, Cândida G. Silva, Filipa Januário, Joana Cruz

**Affiliations:** a Center for Innovative Care and Health Technology (ciTechCare), Polytechnic of Leiria, Leiria, Portugal; b Santa Casa da Misericórdia de Porto de Mós, Leiria, Portugal; c Clínica Albano da Silva Teixeira, Oliveira de Azeméis, Portugal; d Lab3R – Respiratory Research and Rehabilitation Laboratory, School of Health Sciences, University of Aveiro (ESSUA), Aveiro, Portugal; e Institute of Biomedicine (iBiMED), University of Aveiro, Aveiro, Portugal; f Respiratory Medicine, West Park Healthcare Centre, Toronto, Canada; g School of Rehabilitation Sciences, Faculty of Health Sciences, McMaster University, Hamilton, Canada; h School of Health Sciences (ESSLei), Polytechnic of Leiria, Leiria, Portugal; i Coimbra Chemistry Centre, Department of Chemistry, University of Coimbra, Coimbra, Portugal; j Serviço de Medicina Física e de Reabilitação – Centro Hospitalar de Leiria, Leiria, Portugal

## Abstract

**Introduction:**

Chronic obstructive pulmonary disease (COPD) often leads to an increased dependence on the informal carer, which can result in higher levels of distress, anxiety or depression associated with the burden of caregiving and, consequently, reduced quality of life [1]. Several instruments have been used to assess carer burden in COPD; however, their measurement properties have been poorly assessed in this population [2]. The *Questionário de Avaliação de Sobrecarga do Cuidador Informal* (QASCI) is a Portuguese questionnaire, originally created for carers of patients with stroke [3] and later validated in a sample with various chronic diseases, including respiratory diseases [4]. However, its reliability and validity in informal carers of patients with COPD have yet to be established. Therefore, this study aimed to assess the reliability and validity of the QASCI questionnaire for informal carers of patients with COPD.

**Materials and methods:**

The Portuguese questionnaire QASCI evaluates the distress associated with burden of caregiving (scores range from 0 to 100, with higher scores indicating higher levels of burden). It has 32 items divided in 7 subscales (emotional burden; personal life implications; financial burden; reactions to demands; mechanism of efficacy and control; familiar support; and satisfaction with the role). Each item is scored with a 5-point Likert scale. Reliability included internal consistency assessment using the Cronbach’s alpha. Construct validity was assessed using the following questionnaires: Zarit Burden Interview (ZBI) for concurrent validity; the Hospital Anxiety and Depression Scale (HADS) (anxiety and depression) and WHOQOL-Bref (quality of life) for convergent validity. Pearson’s (*r*) or Spearman’s (*ρ*) correlation coefficients were used according to the distribution of each variable. QASCI was expected to present a stronger (positive) correlation with ZBI than with HADS (*r* ≥ 0.5) and a negative correlation with WHOQOL-Bref (*r*≤–0.4) [3,4].

**Results:**

Forty-one carers (62.4 ± 10.1 years, 90.2% female; patients’ FEV_1_=43.7 ± 19.7%pred) completed the questionnaires. Cronbach’s alpha of the full QASCI scale was 0.767 and the subscales presented values between .633 and .929. QASCI and ZBI had a very strong positive correlation (*r* = 0.914; *p*=.01). QASCI had a strong positive correlation with HADS anxiety (*r* = 0.608; *p*=.01) and depression (*ρ* = 0.617; *p*=.01) subscales and moderate to strong negative correlations with all the WHOQOL-Bref subscales (–0.418 to 0.723, *p*=.01).

**Discussion and conclusions:**

QASCI presented good internal consistency and construct validity results. QASCI seems to be a promising measure to evaluate distress levels associated with burden of caregiving in informal carers of patients with COPD.
